# Controlling hemoptysis: An alternative approach

**DOI:** 10.4103/0970-2113.63616

**Published:** 2010

**Authors:** Rakesh K. Chawla, Arun Madan, Dinesh Mehta, Kiran Chawla

**Affiliations:** *Department of Respiratory Medicine, Critical Care and Sleep Disorders, Jaipur Golden Hospital, New Delhi, India*

**Keywords:** Bronchial artery embolization, bronchogenic carcinoma, fiber optic bronchoscopy, hemoptysis

## Abstract

Hemoptysis is a very common symptom in the practice of pulmonary physicians of India. We present a case of uncontrolled hemoptysis managed with bronchial artery embolization. Bronchial artery embolization is an effective treatment for patients with hemoptysis. Serious complications are rare, but may occur if the arterial supply to other structures is compromised.

## INTRODUCTION

Massive hemoptysis usually originates from the systemic arterial supply to the lung. Episodes of hemoptysis may be managed by several approaches, depending on the urgency of the situation, ranging from medical management and bronchial artery embolization (BAE) to surgery. Bronchial artery embolization is a well-accepted and effective form of treatment for massive and recurrent hemoptysis. Bronchial arteries are small vessels that arise directly from the descending thoracic aorta and supply blood to the airways of the lung, esophagus, and lymph nodes.[1‐3] Bronchial arteries show substantial anatomic variations with respect to their origins, branching patterns and courses. The right intercostobronchial trunk, which usually arises from the right posterolateral aspect of the thoracic aorta, is the most constant vessel. Embolization of bronchial arteries is a non-surgical treatment that is safe and effective in patients with massive hemoptysis. The overall immediate success rate ranges between 66.5%[4] to 97%.[5]

## CASE REPORT

A 69-year-old male patient came with complaints of cough and hemoptysis from last one year. The symptoms were not relieved by conservative medications. There was no contact history with any case of pulmonary tuberculosis in the family or neighbourhood. Patient gave no history of any chronic illness, surgery or hospital admission in the past. The patient was a chronic smoker for the past 30 years. The patient was given a trial of anti tubercular treatment for six months but with no relief. Other than crepitations in right inframammary area, rest of the physical examination and routine laboratory work up was inconclusive. Repeated sputa examination for AFB were negative.

**Figure 1:**
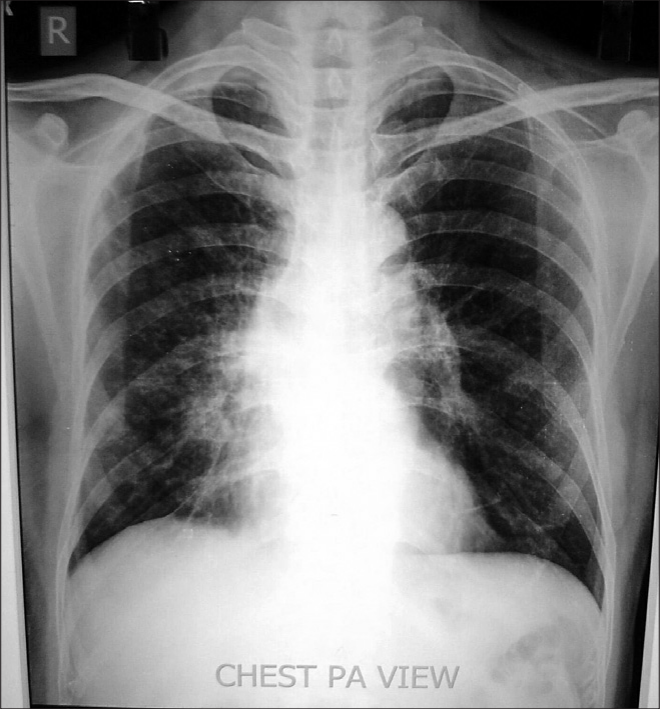
Chest X-ray reveals unfolding of aorta, hazy right parahilar and para cardiac area

**Figure 2:**
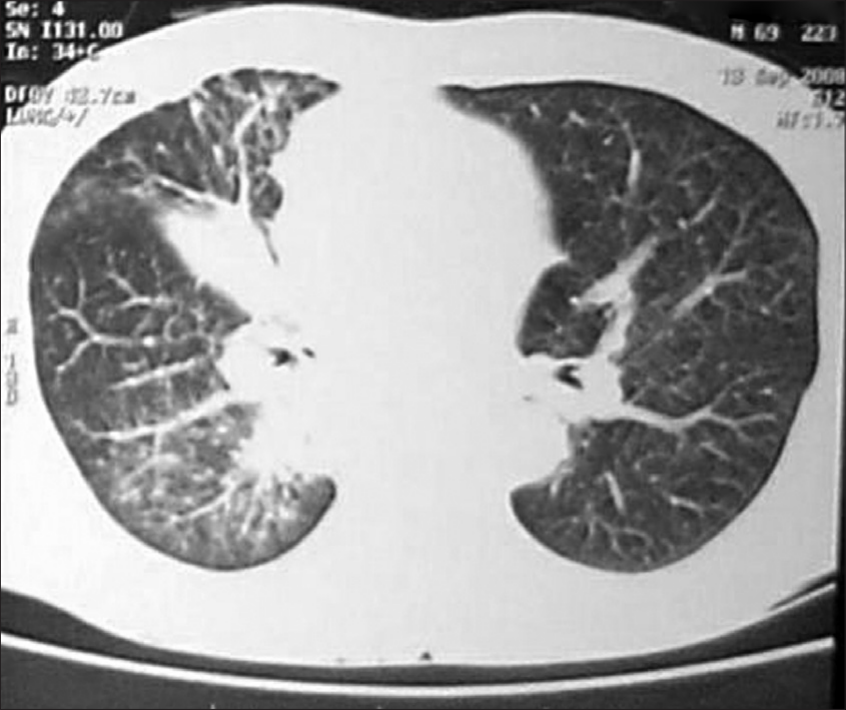
CT chest reveals ill defined soft tissue in the right hilar region encasing bronchus intermedius, causing right middle lobe collapse

**Figure 3:**
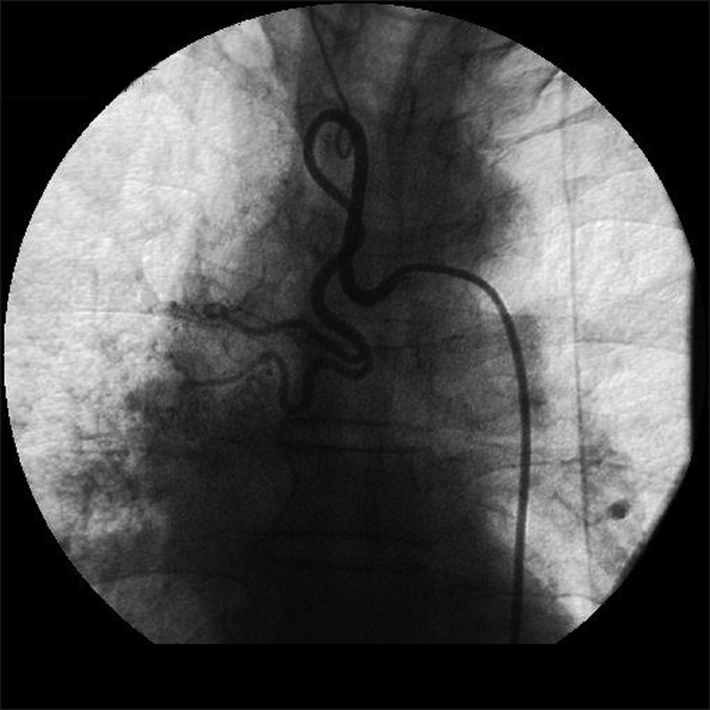
Embolization done at the site of blush

Chest X-ray revealed unfolding of aorta, hazy right parahilar and para cardiac area [Figure 1]. USG whole abdomen and the spirometry were normal. CT chest revealed an ill defined soft tissue opacity in the right hilar region encasing bronchus intermedius and causing right middle lobe collapse and consolidation of superior segment of right lower lobe with mediastinal extension with mediastinal lymphadenopathy and peribronchovascular interstitial thickening raising a suspicion of Bronchogenic carcinoma and Pulmonary tuberculosis [Figure 2]. The case was discussed with the radiologist who advised against bronchial biopsy, keeping in mind the vascular nature of the tumor and the possibility of massive bleed during the bronchial biopsy. Routine bronchoscopy was done to confirm the findings and they revealed that vocal cords, glottis, trachea, carina and left bronchial tree were normal. Right upper lobe was showing pigmented and edematous mucosa. Bronchus Intermedius was so much edematous and swollen which made the openings of right middle lobe and right lower lobe bronchii narrow enough from where the bronchoscope could not be negotiated. It was decided to do bronchial artery embolization to stop the hemoptysis. Bronchial artery embolization was done through the right femoral artery. The procedure involved selective bronchial artery angiography and bronchial artery localization using the contrast omnipaque. Bronchial artery angiography revealed right lung bronchial angiogram showing two bronchial arteries which were dilated and had a blush. Selective angiogram was followed by selective cannulation with micro catheter over bmw wire. Embolization was done with sponge gel [Figure 3]. Hemoptysis stopped immediately after the procedure. Check angiogram revealed closure of the artery. There was no complication. Again fiber optic bronchoscopy was done after one week and biopsy was taken which revealed moderately differentiated squamous cell bronchogenic carcinoma.

## DISCUSSION

Sources of pulmonary bleeding include the low pressure pulmonary circulation (normal pulmonary artery pressure systolic is 15-20 and diastolic 5-10 mm Hg) and the high pressure bronchial circulation (at systemic blood pressure). Bronchial circulation arises from the aorta and therefore has systemic arterial pressure. The bronchial arteries are the main supply of the airways and the supporting structures of the lung while pulmonary arteries supply the lung parenchyma and respiratory bronchioles. Bronchial circulation is the source of massive hemoptysis in 90% of cases.[2]

Arteriographic studies have also demonstrated that the major source of bleeding (92%) is systemic circulation due to necrosis of the wall of bronchial vessels, mucosal ulceration and calcified lymph nodes eroding into the vessels or rupture of Rasmussen aneurysm. More than 80% causes of hemoptysis in patients of bronchogenic carcinoma are due to squamous cell carcinomas. Many researchers currently suggest that CT should be performed prior to bronchoscopy in all cases of massive hemoptysis.[2] The recent introduction of multidetector row CT has offered a comprehensive noninvasive method of evaluating the entire thorax, which allows clear depiction of origins and courses of abnormally dilated bronchial or non-bronchial systemic arteries, which may be the source of the hemorrhage[6] and require embolization.[7,8] The importance of CT can be adjudged by the present case where the radiologist advised not to go for bronchial biopsy without taking the precaution of managing the risk of massive bleeding. Management of patients with life threatening hemoptysis is a therapeutic problem because such patients are often poor surgical candidates.

Bronchial artery embolization for the treatment of hemoptysis was first described in 1973 by Remy *et al*.[9] Additional studies emphasizing the safety and efficacy of bronchial artery embolization followed. A wide range of immediate success rates following BAE have been reported in the literature, ranging between 66.5%[4], 77%[10], 86%[11] and 97%.[5] The probable reason for this discrepancy is selection of patients and super specialty expertise at the Centre, and better recent success rates could be related to more refined techniques and better embolic agents.[2]

Bronchial artery embolization might be the only treatment option available to patients who are not fit to undergo surgery.[12] Bronchial artery embolization (BAE) is a useful and safe alternative procedure in these situations.[13] Many sterile substances are use for embolization, namely Polyvinyl alcohol (PVA),[14,15] Coils, gelform sponge[12] and a new agent, tris-acryl gelatin.[5] Cheng *et al* 2005[5] have used particle size ranging from 300 to 700 um for BAE, so that they could embolize only at the arteriole and capillary levels, while keeping the proximal parent arteries patent. Furthermore, these particles are unlikely to pass through the anterior medullary artery or bronchio-pulmonary shunt.[5]

BAE is an alternative safe and effective nonsurgical treatment for patients with massive hemoptysis which can be done simultaneously on both sides. Success rate is about 88%.[4] Interventional pulmonologist should also be familiar with the possible complications of BAE. Lee *et al* 2008[12] reported a patient with dissection and perforation of vessel with hemo-mediastinum which resolved with time. Spinal cord ischaemia, although a theoretical probability was rarely observed. Cheng *et al* 2005[5] in their retrospective study on BAE also did not observe any major complication like bronchial infarct, stroke or transverse myelitis and there was no procedure related mortality.[5] However, Mal *et al* 1999[10] concluded that BAE may result in long-term as well as immediate control of life threatening hemoptysis but that complications are not unusual. In their series of 56 patients, there were two episodes of mediastinal hematoma which resolved and three episodes of neurologic damage, two of which improved without permanent sequelae but one patient developed paraplegia which did not regress.[10] There have been other sporadic reports of systemic infarcts following BAE, namely renal and splenic infarcts[16], posterior cerebral infarct[17] and Acute MI.[18] Vinaya *et al* 2004[18] reported myocardial infarcts during a BAE with 500 *µ*m Embosphere particles. The authors suggested that the Embosphere particles had crossed the bronchiopulmonary shunt into the systemic circulation. Severe but rare complications are neurological deficiencies and pulmonary necrosis.[14] Neurological deficiencies due to spinal artery embolization are often transient because occlusions are partial. In one report there was only one procedure related complication in 87 patients[4] and in another report, there was only a transient paraparesis in 16 patients.[6] Recurrent bleeding that needs second embolization may occur.[5,10,15] Proximal portion of bronchial arteries should be preserved for second embolization.[19] About 5% of patients may require lobectomy as an emergency procedure.

This case has been presented with the view that in cases of hemoptysis, bronchial biopsy should not be jumped to and the possibility of vascular nature of the tumor should be kept in mind and thoroughly discussed so that if required bronchial artery embolization could be done to control hemoptysis and to avoid massive bleeding during bronchial biopsy.
